# 2-Amino-4-(4-chloro­phen­yl)-6-ferro­cenylpyridine-3-carbonitrile

**DOI:** 10.1107/S1600536808008714

**Published:** 2008-04-26

**Authors:** Jinpeng Zhang, Shu Yan, Zhen He, Lichun Xu, Shuiping Huang

**Affiliations:** a Department of Public Health, Xuzhou Medical College, Xuzhou 221000, People’s Republic of China; b College of Chemistry and Chemical Engineering, Xuzhou Normal University, Xuzhou 221116, People’s Republic of China

## Abstract

In the mol­ecule of the title compound, [Fe(C_5_H_5_)(C_17_H_11_ClN_3_)], the dihedral angles between the two five–membered rings and between the two six-membered rings are 3.28 (4) and 51.33 (4)°, respectively. In the crystal structure, inter­molecular N—H⋯N hydrogen bonds link the mol­ecules into centrosymmetric dimers.

## Related literature

For general background, see: Dombrowski *et al.* (1986 ▶); Alyoubi (2000 ▶); Desai & Shah (2003 ▶); Murata *et al.* (2004 ▶).
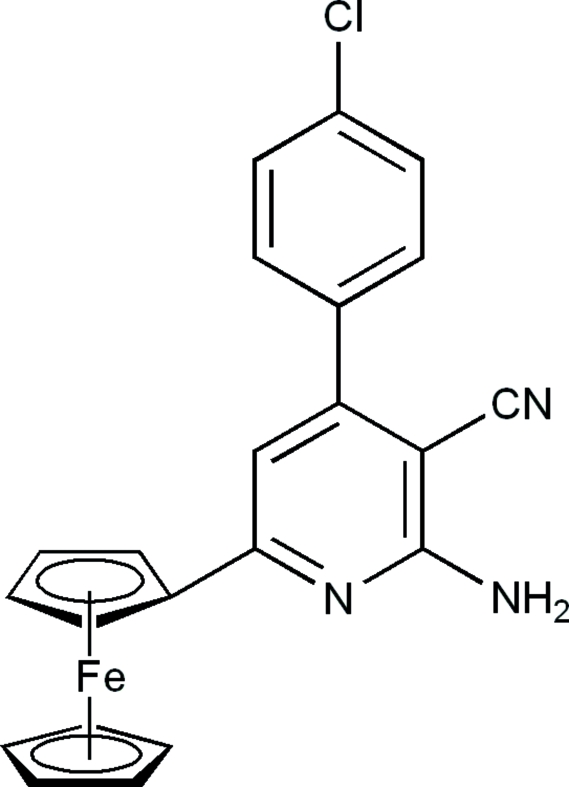


## Experimental

### 

#### Crystal data

[Fe(C_5_H_5_)(C_17_H_11_ClN_3_)]
*M* *_r_* = 413.68
Monoclinic, 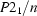

*a* = 12.1517 (13) Å
*b* = 7.4214 (11) Å
*c* = 20.742 (2) Å
β = 97.691 (2)°
*V* = 1853.7 (4) Å^3^
*Z* = 4
Mo *K*α radiation
μ = 0.97 mm^−1^
*T* = 298 (2) K
0.20 × 0.15 × 0.09 mm

#### Data collection

Bruker SMART CCD area-detector diffractometer
Absorption correction: multi-scan (*SADABS*; Sheldrick, 1996 ▶) *T*_min_ = 0.830, *T*_max_ = 0.918
8876 measured reflections
3260 independent reflections
2298 reflections with *I* > 2σ(*I*)
*R*_int_ = 0.041

#### Refinement

*R*[*F* ^2^ > 2σ(*F* ^2^)] = 0.046
*wR*(*F* ^2^) = 0.093
*S* = 1.09
3260 reflections
244 parameters
H-atom parameters constrained
Δρ_max_ = 0.44 e Å^−3^
Δρ_min_ = −0.43 e Å^−3^

### 

Data collection: *SMART* (Bruker, 1998 ▶); cell refinement: *SAINT* (Bruker, 1999 ▶); data reduction: *SAINT*; program(s) used to solve structure: *SHELXS97* (Sheldrick, 2008 ▶); program(s) used to refine structure: *SHELXL97* (Sheldrick, 2008 ▶); molecular graphics: *SHELXTL* (Sheldrick, 2008 ▶); software used to prepare material for publication: *SHELXTL*.

## Supplementary Material

Crystal structure: contains datablocks global, I. DOI: 10.1107/S1600536808008714/hk2443sup1.cif

Structure factors: contains datablocks I. DOI: 10.1107/S1600536808008714/hk2443Isup2.hkl

Additional supplementary materials:  crystallographic information; 3D view; checkCIF report

## Figures and Tables

**Table 1:** Hydrogen-bond geometry (Å, °)

*D*—H⋯*A*	*D*—H	H⋯*A*	*D*⋯*A*	*D*—H⋯*A*
N2—H2*B*⋯N3^i^	0.86	2.28	3.047 (5)	149

Symmetry code: (i) 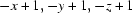.

**Fig. 1.**
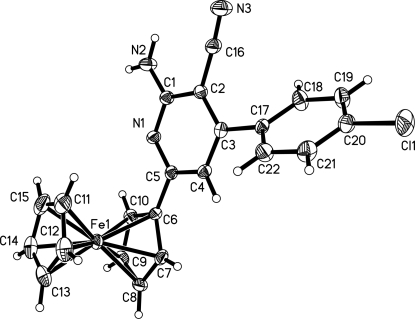
The molecular structure of the title molecule, with the atom-numbering scheme. Displacement ellipsoids are drawn at the 30% probability level.

**Fig. 2.**
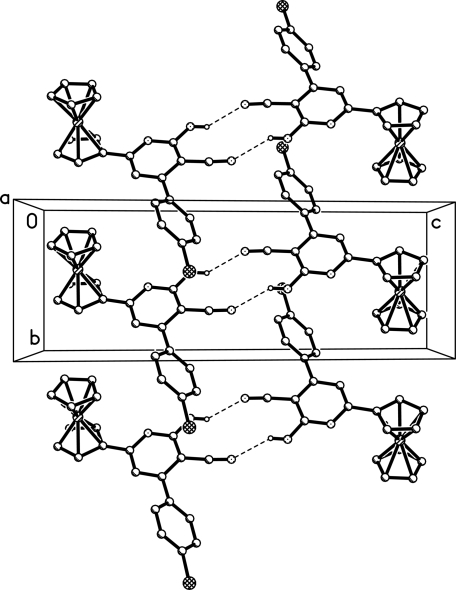
A partial packing diagram of (I). Hydrogen bonds are shown as dashed lines. Hydrogen atoms not involved in hydrogen bonding are omitted for clarity.

## supplementary crystallographic information

### Comment

Metallocenes are known to exhibit a wide range of biological activity. Among them, ferrocene has attracted special attention since it is neutral, chemically stable, non-toxic and able to cross cell membranes (Dombrowski *et al.*, 1986). In fact, it is now well established that the incorporation of ferrocene units into organic molecules introduces significant and new properties in these materials. In addition, it has been demonstrated that molecules containing cyanopyridine moiety may be able to work as ligands towards transition-metal ions (Alyoubi, 2000), new drugs (Murata *et al.*,  2004; Desai & Shah, 2003) and significant intermediates for the synthesis of important vitamins such as nicotinic acids and nicotinamides. For these reasons, the synthesis of new compounds containing cyanopyridine derivatives is strongly desired. We report herein the crystal structure of the title compound, (I).

In the molecule of (I), (Fig. 1), rings A (N1/C1-C5), B (C17-C22), C (C6-C10) and D (C11-C15) are, of course, planar. The dihedral angles between them are A/B = 51.33 (4)°, A/C = 13.20 (3)°, A/D = 16.32 (4)°, B/C = 46.38 (3)°, B/D= 44.47 (3)° and C/D = 3.28 (4)°. So, rings C and D are nearly paralllel to each other.

In the crystal structure, intermolecular N-H···N hydrogen bonds (Table 1) link the molecules into centrosymmetric dimers (Fig. 2), in which they may be effective in the stabilization of the structure.

### Experimental

Compound (I) was prepared by the reaction of 4-chlorobenzaldehyde (2 mmol), malononitrile (2 mmol), acetylferrocene (2 mmol) and ammonium acetate (4 mmol) in water (2 ml). Single crystals of (I) suitable for X-ray analysis were obtained by slow evaporation of an aqueous ethanol solution (95%) (yield; 95%, m.p. 548-550 K). IR (cm^-1^): 3457, 3354, 2211; ^1^H NMR (DMSO-d_6_): 4.10 (5*H*, s, ferrocenyl), 4.50 (2*H*, s, ferrocenyl), 5.04 (2*H*, s, ferrocenyl), 6.81 (2*H*, brs, NH_2_), 6.95 (1*H*, s, ArH), 7.64 (2*H*, d, J = 8.4 Hz, ArH), 7.68 (2*H*, d, J = 8.4 Hz, ArH).7.87 (2*H*, brs, NH_2_), 7.88–8.01 (4*H*, m, ArH), 11.85 (1*H*, s, NH).

### Refinement

H atoms were positioned geometrically, with N-H = 0.86 Å (for NH_2_) and C-H = 0.93 Å for aromatic H,and constrained to ride on their parent atoms with U_iso_(H) = 1.2U_eq_(C,N).

### Crystal data

**Table:**

[Fe(C_5_H_5_)(C_17_H_11_ClN_3_)]	*F*_000_ = 848
*M**_r_* = 413.68	*D*_x_ = 1.482 Mg m^−^^3^
Monoclinic, *P*2_1_/*n*	Melting point = 548–550 K
Hall symbol: -P 2yn	Mo *K*α radiation λ = 0.71073 Å
*a* = 12.1517 (13) Å	Cell parameters from 2405 reflections
*b* = 7.4214 (11) Å	θ = 2.9–26.2º
*c* = 20.742 (2) Å	µ = 0.97 mm^−^^1^
β = 97.691 (2)º	*T* = 298 (2) K
*V* = 1853.7 (4) Å^3^	Block, red
*Z* = 4	0.20 × 0.15 × 0.09 mm

### Data collection

**Table:**

Bruker SMART CCD area-detector diffractometer	3260 independent reflections
Radiation source: fine-focus sealed tube	2298 reflections with *I* > 2σ(*I*)
Monochromator: graphite	*R*_int_ = 0.041
*T* = 298(2) K	θ_max_ = 25.0º
φ and ω scans	θ_min_ = 1.8º
Absorption correction: multi-scan(SADABS; Sheldrick, 1996)	*h* = −14→14
*T*_min_ = 0.830, *T*_max_ = 0.918	*k* = −8→6
8876 measured reflections	*l* = −24→24

### Refinement

**Table:**

Refinement on *F*^2^	Secondary atom site location: difference Fourier map
Least-squares matrix: full	Hydrogen site location: inferred from neighbouring sites
*R*[*F*^2^ > 2σ(*F*^2^)] = 0.046	H-atom parameters constrained
*wR*(*F*^2^) = 0.093	*w* = 1/[σ^2^(*F*_o_^2^) + (0.0178*P*)^2^ + 2.3504*P*] where *P* = (*F*_o_^2^ + 2*F*_c_^2^)/3
*S* = 1.09	(Δ/σ)_max_ < 0.001
3260 reflections	Δρ_max_ = 0.44 e Å^−^^3^
244 parameters	Δρ_min_ = −0.43 e Å^−^^3^
Primary atom site location: structure-invariant direct methods	Extinction correction: none

### Special details

**Table:**

Geometry. All e.s.d.'s (except the e.s.d. in the dihedral angle between two l.s. planes) are estimated using the full covariance matrix. The cell e.s.d.'s are taken into account individually in the estimation of e.s.d.'s in distances, angles and torsion angles; correlations between e.s.d.'s in cell parameters are only used when they are defined by crystal symmetry. An approximate (isotropic) treatment of cell e.s.d.'s is used for estimating e.s.d.'s involving l.s. planes.
Refinement. Refinement of F^2^ against ALL reflections. The weighted R-factor wR and goodness of fit S are based on F^2^, conventional R-factors R are based on F, with F set to zero for negative F^2^. The threshold expression of F^2^ > 2sigma(F^2^) is used only for calculating R-factors(gt) etc. and is not relevant to the choice of reflections for refinement. R-factors based on F^2^ are statistically about twice as large as those based on F, and R- factors based on ALL data will be even larger.

### Fractional atomic coordinates and isotropic or equivalent isotropic displacement parameters (Å^2^)

**Table:**

	*x*	*y*	*z*	*U*_iso_*/*U*_eq_	
Fe1	0.38223 (4)	0.42499 (7)	0.10732 (3)	0.03424 (16)	
Cl1	0.81792 (10)	1.44271 (17)	0.38672 (6)	0.0694 (4)	
N1	0.3585 (2)	0.5432 (4)	0.27711 (13)	0.0335 (7)	
N2	0.3644 (2)	0.4443 (4)	0.38140 (14)	0.0450 (8)	
H2A	0.3154	0.3649	0.3672	0.054*	
H2B	0.3892	0.4491	0.4222	0.054*	
N3	0.5500 (3)	0.6983 (5)	0.48341 (17)	0.0652 (12)	
C1	0.4019 (3)	0.5604 (5)	0.33992 (16)	0.0321 (8)	
C2	0.4807 (3)	0.6949 (5)	0.36089 (16)	0.0301 (8)	
C3	0.5168 (3)	0.8132 (5)	0.31540 (17)	0.0307 (8)	
C4	0.4739 (3)	0.7885 (5)	0.25086 (17)	0.0309 (8)	
H4	0.4977	0.8613	0.2189	0.037*	
C5	0.3950 (3)	0.6543 (5)	0.23368 (16)	0.0292 (8)	
C6	0.3441 (3)	0.6283 (5)	0.16610 (16)	0.0287 (8)	
C7	0.3802 (3)	0.6998 (5)	0.10848 (18)	0.0366 (9)	
H7	0.4408	0.7755	0.1070	0.044*	
C8	0.3080 (3)	0.6355 (5)	0.05402 (17)	0.0396 (10)	
H8	0.3125	0.6617	0.0106	0.048*	
C9	0.2277 (3)	0.5243 (5)	0.07769 (17)	0.0390 (10)	
H9	0.1701	0.4648	0.0523	0.047*	
C10	0.2493 (3)	0.5186 (5)	0.14592 (17)	0.0345 (9)	
H10	0.2088	0.4543	0.1732	0.041*	
C11	0.5039 (4)	0.2760 (6)	0.1590 (2)	0.0615 (13)	
H11	0.5385	0.3005	0.2008	0.074*	
C12	0.5390 (3)	0.3359 (6)	0.1002 (2)	0.0582 (13)	
H12	0.6003	0.4081	0.0964	0.070*	
C13	0.4640 (4)	0.2662 (6)	0.0484 (2)	0.0562 (12)	
H13	0.4672	0.2835	0.0042	0.067*	
C14	0.3841 (4)	0.1665 (6)	0.0753 (3)	0.0594 (13)	
H14	0.3249	0.1057	0.0519	0.071*	
C15	0.4071 (4)	0.1726 (6)	0.1426 (3)	0.0648 (14)	
H15	0.3658	0.1178	0.1717	0.078*	
C16	0.5217 (3)	0.7025 (5)	0.42883 (19)	0.0409 (10)	
C17	0.5934 (3)	0.9656 (5)	0.33533 (17)	0.0308 (8)	
C18	0.5737 (3)	1.0847 (6)	0.38359 (19)	0.0469 (10)	
H18	0.5127	1.0663	0.4055	0.056*	
C19	0.6424 (3)	1.2308 (6)	0.4002 (2)	0.0489 (11)	
H19	0.6276	1.3099	0.4328	0.059*	
C20	0.7325 (3)	1.2576 (5)	0.36813 (19)	0.0429 (10)	
C21	0.7559 (3)	1.1393 (6)	0.3206 (2)	0.0487 (11)	
H21	0.8182	1.1567	0.2998	0.058*	
C22	0.6861 (3)	0.9946 (6)	0.30423 (19)	0.0450 (10)	
H22	0.7015	0.9155	0.2718	0.054*	

### Atomic displacement parameters (Å^2^)

**Table:**

	*U*^11^	*U*^22^	*U*^33^	*U*^12^	*U*^13^	*U*^23^
Fe1	0.0363 (3)	0.0281 (3)	0.0382 (3)	0.0077 (3)	0.0046 (2)	−0.0010 (3)
Cl1	0.0680 (7)	0.0544 (8)	0.0859 (9)	−0.0359 (6)	0.0103 (6)	−0.0084 (7)
N1	0.0387 (16)	0.0311 (18)	0.0301 (15)	−0.0057 (15)	0.0022 (13)	0.0036 (15)
N2	0.0533 (19)	0.049 (2)	0.0304 (16)	−0.0262 (18)	−0.0027 (14)	0.0062 (18)
N3	0.085 (3)	0.067 (3)	0.039 (2)	−0.033 (2)	−0.0095 (19)	0.010 (2)
C1	0.0330 (18)	0.033 (2)	0.0308 (19)	−0.0026 (18)	0.0045 (15)	0.0014 (19)
C2	0.0306 (19)	0.029 (2)	0.0293 (19)	−0.0047 (16)	0.0005 (16)	−0.0001 (17)
C3	0.0280 (18)	0.027 (2)	0.038 (2)	−0.0002 (16)	0.0048 (16)	−0.0007 (17)
C4	0.0320 (19)	0.029 (2)	0.032 (2)	−0.0036 (16)	0.0054 (16)	0.0039 (17)
C5	0.0295 (18)	0.0260 (19)	0.0326 (19)	0.0050 (16)	0.0056 (16)	0.0004 (17)
C6	0.0323 (19)	0.027 (2)	0.0264 (18)	0.0038 (16)	0.0022 (15)	0.0028 (16)
C7	0.042 (2)	0.026 (2)	0.042 (2)	0.0062 (19)	0.0067 (18)	0.006 (2)
C8	0.050 (2)	0.042 (2)	0.0275 (19)	0.011 (2)	0.0043 (18)	0.0011 (19)
C9	0.039 (2)	0.040 (2)	0.036 (2)	0.0075 (18)	−0.0011 (17)	−0.0050 (19)
C10	0.0318 (19)	0.035 (2)	0.036 (2)	0.0041 (17)	0.0018 (16)	−0.0006 (18)
C11	0.065 (3)	0.054 (3)	0.061 (3)	0.031 (3)	−0.008 (3)	0.001 (3)
C12	0.039 (2)	0.052 (3)	0.084 (4)	0.018 (2)	0.006 (2)	−0.009 (3)
C13	0.058 (3)	0.049 (3)	0.064 (3)	0.014 (2)	0.017 (2)	−0.017 (3)
C14	0.065 (3)	0.033 (3)	0.079 (4)	0.007 (2)	0.006 (3)	−0.011 (3)
C15	0.075 (3)	0.038 (3)	0.083 (4)	0.017 (3)	0.017 (3)	0.015 (3)
C16	0.048 (2)	0.036 (2)	0.037 (2)	−0.0157 (19)	0.0013 (19)	0.006 (2)
C17	0.0309 (18)	0.026 (2)	0.0358 (19)	−0.0052 (16)	0.0041 (15)	0.0030 (17)
C18	0.043 (2)	0.044 (2)	0.057 (3)	−0.014 (2)	0.0189 (19)	−0.012 (2)
C19	0.053 (3)	0.040 (2)	0.056 (3)	−0.011 (2)	0.013 (2)	−0.012 (2)
C20	0.043 (2)	0.039 (2)	0.046 (2)	−0.014 (2)	0.0060 (19)	0.000 (2)
C21	0.041 (2)	0.049 (3)	0.060 (3)	−0.013 (2)	0.019 (2)	0.002 (2)
C22	0.045 (2)	0.045 (2)	0.046 (2)	−0.008 (2)	0.0123 (19)	−0.004 (2)

### Geometric parameters (Å, °)

**Table:**

Fe1—C15	2.019 (5)	C7—C8	1.417 (5)
Fe1—C10	2.020 (3)	C7—H7	0.9300
Fe1—C14	2.031 (4)	C8—C9	1.415 (5)
Fe1—C6	2.032 (3)	C8—H8	0.9300
Fe1—C11	2.033 (4)	C9—C10	1.405 (5)
Fe1—C9	2.034 (4)	C9—H9	0.9300
Fe1—C7	2.040 (4)	C10—H10	0.9300
Fe1—C12	2.040 (4)	C11—C15	1.408 (6)
Fe1—C13	2.048 (4)	C11—C12	1.415 (6)
Fe1—C8	2.052 (4)	C11—H11	0.9300
Cl1—C20	1.734 (4)	C12—C13	1.412 (6)
N1—C5	1.339 (4)	C12—H12	0.9300
N1—C1	1.344 (4)	C13—C14	1.395 (6)
N2—C1	1.340 (4)	C13—H13	0.9300
N2—H2A	0.8600	C14—C15	1.385 (6)
N2—H2B	0.8600	C14—H14	0.9300
N3—C16	1.139 (4)	C15—H15	0.9300
C1—C2	1.411 (5)	C17—C18	1.380 (5)
C2—C3	1.401 (5)	C17—C22	1.388 (5)
C2—C16	1.432 (5)	C18—C19	1.384 (5)
C3—C4	1.382 (5)	C18—H18	0.9300
C3—C17	1.488 (5)	C19—C20	1.369 (5)
C4—C5	1.396 (5)	C19—H19	0.9300
C4—H4	0.9300	C20—C21	1.377 (5)
C5—C6	1.467 (4)	C21—C22	1.383 (5)
C6—C10	1.426 (5)	C21—H21	0.9300
C6—C7	1.429 (5)	C22—H22	0.9300
			
C15—Fe1—C10	105.34 (18)	C6—C7—Fe1	69.2 (2)
C15—Fe1—C14	40.01 (18)	C8—C7—H7	125.8
C10—Fe1—C14	119.93 (18)	C6—C7—H7	125.8
C15—Fe1—C6	120.45 (18)	Fe1—C7—H7	126.4
C10—Fe1—C6	41.22 (13)	C9—C8—C7	107.6 (3)
C14—Fe1—C6	155.60 (18)	C9—C8—Fe1	69.1 (2)
C15—Fe1—C11	40.67 (18)	C7—C8—Fe1	69.3 (2)
C10—Fe1—C11	122.83 (18)	C9—C8—H8	126.2
C14—Fe1—C11	67.62 (19)	C7—C8—H8	126.2
C6—Fe1—C11	107.21 (16)	Fe1—C8—H8	127.0
C15—Fe1—C9	122.21 (19)	C10—C9—C8	108.8 (3)
C10—Fe1—C9	40.55 (13)	C10—C9—Fe1	69.2 (2)
C14—Fe1—C9	107.10 (17)	C8—C9—Fe1	70.4 (2)
C6—Fe1—C9	68.69 (14)	C10—C9—H9	125.6
C11—Fe1—C9	158.94 (19)	C8—C9—H9	125.6
C15—Fe1—C7	157.7 (2)	Fe1—C9—H9	126.4
C10—Fe1—C7	68.88 (15)	C9—C10—C6	108.3 (3)
C14—Fe1—C7	161.62 (19)	C9—C10—Fe1	70.3 (2)
C6—Fe1—C7	41.10 (13)	C6—C10—Fe1	69.86 (19)
C11—Fe1—C7	123.14 (18)	C9—C10—H10	125.9
C9—Fe1—C7	68.23 (15)	C6—C10—H10	125.9
C15—Fe1—C12	68.25 (19)	Fe1—C10—H10	125.6
C10—Fe1—C12	160.75 (17)	C15—C11—C12	107.5 (4)
C14—Fe1—C12	67.58 (19)	C15—C11—Fe1	69.1 (2)
C6—Fe1—C12	125.06 (16)	C12—C11—Fe1	69.9 (2)
C11—Fe1—C12	40.67 (17)	C15—C11—H11	126.2
C9—Fe1—C12	158.22 (17)	C12—C11—H11	126.2
C7—Fe1—C12	109.75 (18)	Fe1—C11—H11	126.3
C15—Fe1—C13	67.7 (2)	C13—C12—C11	107.6 (4)
C10—Fe1—C13	155.78 (16)	C13—C12—Fe1	70.1 (2)
C14—Fe1—C13	40.00 (17)	C11—C12—Fe1	69.4 (2)
C6—Fe1—C13	162.41 (16)	C13—C12—H12	126.2
C11—Fe1—C13	67.98 (19)	C11—C12—H12	126.2
C9—Fe1—C13	122.00 (16)	Fe1—C12—H12	125.9
C7—Fe1—C13	126.16 (18)	C14—C13—C12	107.5 (4)
C12—Fe1—C13	40.39 (16)	C14—C13—Fe1	69.3 (2)
C15—Fe1—C8	159.28 (19)	C12—C13—Fe1	69.5 (2)
C10—Fe1—C8	68.55 (15)	C14—C13—H13	126.2
C14—Fe1—C8	124.52 (18)	C12—C13—H13	126.2
C6—Fe1—C8	68.82 (13)	Fe1—C13—H13	126.5
C11—Fe1—C8	159.14 (19)	C15—C14—C13	109.2 (5)
C9—Fe1—C8	40.52 (14)	C15—C14—Fe1	69.5 (3)
C7—Fe1—C8	40.53 (14)	C13—C14—Fe1	70.7 (3)
C12—Fe1—C8	123.74 (18)	C15—C14—H14	125.4
C13—Fe1—C8	109.26 (17)	C13—C14—H14	125.4
C5—N1—C1	118.1 (3)	Fe1—C14—H14	126.0
C1—N2—H2A	120.0	C14—C15—C11	108.1 (5)
C1—N2—H2B	120.0	C14—C15—Fe1	70.5 (3)
H2A—N2—H2B	120.0	C11—C15—Fe1	70.2 (3)
N2—C1—N1	116.0 (3)	C14—C15—H15	126.0
N2—C1—C2	122.2 (3)	C11—C15—H15	126.0
N1—C1—C2	121.8 (3)	Fe1—C15—H15	125.0
C3—C2—C1	119.8 (3)	N3—C16—C2	175.2 (4)
C3—C2—C16	122.6 (3)	C18—C17—C22	117.9 (3)
C1—C2—C16	117.6 (3)	C18—C17—C3	121.7 (3)
C4—C3—C2	117.2 (3)	C22—C17—C3	120.4 (3)
C4—C3—C17	120.7 (3)	C17—C18—C19	121.7 (4)
C2—C3—C17	122.0 (3)	C17—C18—H18	119.2
C3—C4—C5	119.9 (3)	C19—C18—H18	119.2
C3—C4—H4	120.0	C20—C19—C18	119.2 (4)
C5—C4—H4	120.0	C20—C19—H19	120.4
N1—C5—C4	123.1 (3)	C18—C19—H19	120.4
N1—C5—C6	115.2 (3)	C19—C20—C21	120.7 (4)
C4—C5—C6	121.7 (3)	C19—C20—Cl1	120.0 (3)
C10—C6—C7	107.0 (3)	C21—C20—Cl1	119.3 (3)
C10—C6—C5	125.2 (3)	C20—C21—C22	119.5 (4)
C7—C6—C5	127.8 (3)	C20—C21—H21	120.3
C10—C6—Fe1	68.9 (2)	C22—C21—H21	120.3
C7—C6—Fe1	69.73 (19)	C21—C22—C17	121.0 (4)
C5—C6—Fe1	124.7 (2)	C21—C22—H22	119.5
C8—C7—C6	108.4 (3)	C17—C22—H22	119.5
C8—C7—Fe1	70.2 (2)		
			
C5—N1—C1—N2	−178.8 (3)	C13—Fe1—C10—C9	−52.3 (5)
C5—N1—C1—C2	2.2 (5)	C8—Fe1—C10—C9	37.1 (2)
N2—C1—C2—C3	−179.8 (3)	C15—Fe1—C10—C6	119.0 (2)
N1—C1—C2—C3	−0.9 (5)	C14—Fe1—C10—C6	159.6 (2)
N2—C1—C2—C16	0.9 (5)	C11—Fe1—C10—C6	78.3 (3)
N1—C1—C2—C16	179.8 (3)	C9—Fe1—C10—C6	−119.1 (3)
C1—C2—C3—C4	−1.4 (5)	C7—Fe1—C10—C6	−38.3 (2)
C16—C2—C3—C4	177.8 (3)	C12—Fe1—C10—C6	51.3 (6)
C1—C2—C3—C17	175.5 (3)	C13—Fe1—C10—C6	−171.4 (4)
C16—C2—C3—C17	−5.3 (5)	C8—Fe1—C10—C6	−81.9 (2)
C2—C3—C4—C5	2.3 (5)	C10—Fe1—C11—C15	74.5 (3)
C17—C3—C4—C5	−174.6 (3)	C14—Fe1—C11—C15	−37.6 (3)
C1—N1—C5—C4	−1.3 (5)	C6—Fe1—C11—C15	117.0 (3)
C1—N1—C5—C6	−179.9 (3)	C9—Fe1—C11—C15	41.7 (6)
C3—C4—C5—N1	−1.1 (5)	C7—Fe1—C11—C15	159.3 (3)
C3—C4—C5—C6	177.5 (3)	C12—Fe1—C11—C15	−118.8 (4)
N1—C5—C6—C10	10.9 (5)	C13—Fe1—C11—C15	−81.0 (3)
C4—C5—C6—C10	−167.8 (3)	C8—Fe1—C11—C15	−167.5 (4)
N1—C5—C6—C7	−166.7 (3)	C15—Fe1—C11—C12	118.8 (4)
C4—C5—C6—C7	14.6 (6)	C10—Fe1—C11—C12	−166.7 (3)
N1—C5—C6—Fe1	−76.5 (4)	C14—Fe1—C11—C12	81.2 (3)
C4—C5—C6—Fe1	104.9 (4)	C6—Fe1—C11—C12	−124.2 (3)
C15—Fe1—C6—C10	−78.2 (3)	C9—Fe1—C11—C12	160.5 (4)
C14—Fe1—C6—C10	−46.9 (5)	C7—Fe1—C11—C12	−81.9 (3)
C11—Fe1—C6—C10	−120.5 (2)	C13—Fe1—C11—C12	37.8 (3)
C9—Fe1—C6—C10	37.6 (2)	C8—Fe1—C11—C12	−48.6 (6)
C7—Fe1—C6—C10	118.5 (3)	C15—C11—C12—C13	−0.8 (5)
C12—Fe1—C6—C10	−161.7 (2)	Fe1—C11—C12—C13	−59.9 (3)
C13—Fe1—C6—C10	168.2 (5)	C15—C11—C12—Fe1	59.1 (3)
C8—Fe1—C6—C10	81.2 (2)	C15—Fe1—C12—C13	80.8 (3)
C15—Fe1—C6—C7	163.4 (2)	C10—Fe1—C12—C13	154.6 (5)
C10—Fe1—C6—C7	−118.5 (3)	C14—Fe1—C12—C13	37.4 (3)
C14—Fe1—C6—C7	−165.4 (4)	C6—Fe1—C12—C13	−166.5 (3)
C11—Fe1—C6—C7	121.0 (3)	C11—Fe1—C12—C13	118.7 (4)
C9—Fe1—C6—C7	−80.9 (2)	C9—Fe1—C12—C13	−42.5 (6)
C12—Fe1—C6—C7	79.9 (3)	C7—Fe1—C12—C13	−123.0 (3)
C13—Fe1—C6—C7	49.8 (6)	C8—Fe1—C12—C13	−80.0 (3)
C8—Fe1—C6—C7	−37.3 (2)	C15—Fe1—C12—C11	−37.9 (3)
C15—Fe1—C6—C5	40.8 (4)	C10—Fe1—C12—C11	35.9 (7)
C10—Fe1—C6—C5	118.9 (4)	C14—Fe1—C12—C11	−81.3 (3)
C14—Fe1—C6—C5	72.1 (5)	C6—Fe1—C12—C11	74.8 (3)
C11—Fe1—C6—C5	−1.6 (4)	C9—Fe1—C12—C11	−161.2 (4)
C9—Fe1—C6—C5	156.5 (3)	C7—Fe1—C12—C11	118.3 (3)
C7—Fe1—C6—C5	−122.6 (4)	C13—Fe1—C12—C11	−118.7 (4)
C12—Fe1—C6—C5	−42.7 (4)	C8—Fe1—C12—C11	161.3 (3)
C13—Fe1—C6—C5	−72.8 (6)	C11—C12—C13—C14	0.4 (5)
C8—Fe1—C6—C5	−159.8 (3)	Fe1—C12—C13—C14	−59.1 (3)
C10—C6—C7—C8	0.4 (4)	C11—C12—C13—Fe1	59.5 (3)
C5—C6—C7—C8	178.3 (3)	C15—Fe1—C13—C14	36.9 (3)
Fe1—C6—C7—C8	59.5 (3)	C10—Fe1—C13—C14	−40.8 (6)
C10—C6—C7—Fe1	−59.1 (2)	C6—Fe1—C13—C14	158.3 (5)
C5—C6—C7—Fe1	118.8 (4)	C11—Fe1—C13—C14	81.0 (3)
C15—Fe1—C7—C8	−160.1 (4)	C9—Fe1—C13—C14	−78.2 (3)
C10—Fe1—C7—C8	−81.3 (2)	C7—Fe1—C13—C14	−163.3 (3)
C14—Fe1—C7—C8	41.0 (6)	C12—Fe1—C13—C14	119.0 (4)
C6—Fe1—C7—C8	−119.7 (3)	C8—Fe1—C13—C14	−121.2 (3)
C11—Fe1—C7—C8	162.5 (2)	C15—Fe1—C13—C12	−82.1 (3)
C9—Fe1—C7—C8	−37.6 (2)	C10—Fe1—C13—C12	−159.8 (4)
C12—Fe1—C7—C8	119.2 (2)	C14—Fe1—C13—C12	−119.0 (4)
C13—Fe1—C7—C8	76.9 (3)	C6—Fe1—C13—C12	39.3 (7)
C15—Fe1—C7—C6	−40.4 (5)	C11—Fe1—C13—C12	−38.1 (3)
C10—Fe1—C7—C6	38.39 (19)	C9—Fe1—C13—C12	162.8 (3)
C14—Fe1—C7—C6	160.7 (5)	C7—Fe1—C13—C12	77.7 (3)
C11—Fe1—C7—C6	−77.8 (3)	C8—Fe1—C13—C12	119.8 (3)
C9—Fe1—C7—C6	82.1 (2)	C12—C13—C14—C15	0.2 (5)
C12—Fe1—C7—C6	−121.1 (2)	Fe1—C13—C14—C15	−59.0 (3)
C13—Fe1—C7—C6	−163.4 (2)	C12—C13—C14—Fe1	59.2 (3)
C8—Fe1—C7—C6	119.7 (3)	C10—Fe1—C14—C15	−77.8 (3)
C6—C7—C8—C9	−0.2 (4)	C6—Fe1—C14—C15	−44.1 (5)
Fe1—C7—C8—C9	58.6 (3)	C11—Fe1—C14—C15	38.3 (3)
C6—C7—C8—Fe1	−58.8 (2)	C9—Fe1—C14—C15	−120.1 (3)
C15—Fe1—C8—C9	39.2 (6)	C7—Fe1—C14—C15	167.7 (5)
C10—Fe1—C8—C9	−37.2 (2)	C12—Fe1—C14—C15	82.4 (3)
C14—Fe1—C8—C9	75.2 (3)	C13—Fe1—C14—C15	120.2 (4)
C6—Fe1—C8—C9	−81.6 (2)	C8—Fe1—C14—C15	−161.1 (3)
C11—Fe1—C8—C9	−164.4 (4)	C15—Fe1—C14—C13	−120.2 (4)
C7—Fe1—C8—C9	−119.3 (3)	C10—Fe1—C14—C13	162.0 (3)
C12—Fe1—C8—C9	159.6 (2)	C6—Fe1—C14—C13	−164.3 (3)
C13—Fe1—C8—C9	117.1 (2)	C11—Fe1—C14—C13	−82.0 (3)
C15—Fe1—C8—C7	158.6 (5)	C9—Fe1—C14—C13	119.7 (3)
C10—Fe1—C8—C7	82.2 (2)	C7—Fe1—C14—C13	47.5 (7)
C14—Fe1—C8—C7	−165.5 (3)	C12—Fe1—C14—C13	−37.8 (3)
C6—Fe1—C8—C7	37.8 (2)	C8—Fe1—C14—C13	78.6 (3)
C11—Fe1—C8—C7	−45.0 (6)	C13—C14—C15—C11	−0.7 (5)
C9—Fe1—C8—C7	119.3 (3)	Fe1—C14—C15—C11	−60.4 (3)
C12—Fe1—C8—C7	−81.0 (3)	C13—C14—C15—Fe1	59.7 (3)
C13—Fe1—C8—C7	−123.6 (2)	C12—C11—C15—C14	0.9 (5)
C7—C8—C9—C10	−0.1 (4)	Fe1—C11—C15—C14	60.6 (3)
Fe1—C8—C9—C10	58.7 (3)	C12—C11—C15—Fe1	−59.6 (3)
C7—C8—C9—Fe1	−58.8 (3)	C10—Fe1—C15—C14	118.6 (3)
C15—Fe1—C9—C10	75.2 (3)	C6—Fe1—C15—C14	160.5 (3)
C14—Fe1—C9—C10	116.3 (3)	C11—Fe1—C15—C14	−118.5 (4)
C6—Fe1—C9—C10	−38.2 (2)	C9—Fe1—C15—C14	77.9 (3)
C11—Fe1—C9—C10	44.4 (6)	C7—Fe1—C15—C14	−169.8 (4)
C7—Fe1—C9—C10	−82.5 (2)	C12—Fe1—C15—C14	−80.6 (3)
C12—Fe1—C9—C10	−171.5 (4)	C13—Fe1—C15—C14	−36.9 (3)
C13—Fe1—C9—C10	157.5 (2)	C8—Fe1—C15—C14	48.8 (6)
C8—Fe1—C9—C10	−120.1 (3)	C10—Fe1—C15—C11	−122.9 (3)
C15—Fe1—C9—C8	−164.7 (2)	C14—Fe1—C15—C11	118.5 (4)
C10—Fe1—C9—C8	120.1 (3)	C6—Fe1—C15—C11	−80.9 (3)
C14—Fe1—C9—C8	−123.5 (2)	C9—Fe1—C15—C11	−163.6 (3)
C6—Fe1—C9—C8	81.9 (2)	C7—Fe1—C15—C11	−51.3 (6)
C11—Fe1—C9—C8	164.5 (4)	C12—Fe1—C15—C11	37.9 (3)
C7—Fe1—C9—C8	37.6 (2)	C13—Fe1—C15—C11	81.7 (3)
C12—Fe1—C9—C8	−51.3 (5)	C8—Fe1—C15—C11	167.4 (4)
C13—Fe1—C9—C8	−82.4 (3)	C4—C3—C17—C18	126.9 (4)
C8—C9—C10—C6	0.4 (4)	C2—C3—C17—C18	−49.8 (5)
Fe1—C9—C10—C6	59.8 (2)	C4—C3—C17—C22	−51.3 (5)
C8—C9—C10—Fe1	−59.4 (3)	C2—C3—C17—C22	131.9 (4)
C7—C6—C10—C9	−0.5 (4)	C22—C17—C18—C19	1.1 (6)
C5—C6—C10—C9	−178.5 (3)	C3—C17—C18—C19	−177.2 (4)
Fe1—C6—C10—C9	−60.1 (2)	C17—C18—C19—C20	−0.2 (6)
C7—C6—C10—Fe1	59.6 (2)	C18—C19—C20—C21	−1.1 (6)
C5—C6—C10—Fe1	−118.4 (3)	C18—C19—C20—Cl1	178.5 (3)
C15—Fe1—C10—C9	−122.0 (3)	C19—C20—C21—C22	1.5 (6)
C14—Fe1—C10—C9	−81.3 (3)	Cl1—C20—C21—C22	−178.0 (3)
C6—Fe1—C10—C9	119.1 (3)	C20—C21—C22—C17	−0.7 (6)
C11—Fe1—C10—C9	−162.6 (3)	C18—C17—C22—C21	−0.6 (6)
C7—Fe1—C10—C9	80.8 (2)	C3—C17—C22—C21	177.7 (3)
C12—Fe1—C10—C9	170.4 (5)		

### Hydrogen-bond geometry (Å, °)

**Table:**

*D*—H···*A*	*D*—H	H···*A*	*D*···*A*	*D*—H···*A*
N2—H2B···N3^i^	0.86	2.28	3.047 (5)	149

Symmetry codes: (i) −*x*+1, −*y*+1, −*z*+1.
